# Real-Time Estimation of Aerobic Threshold and Exercise Intensity Distribution Using Fractal Correlation Properties of Heart Rate Variability: A Single-Case Field Application in a Former Olympic Triathlete

**DOI:** 10.3389/fspor.2021.668812

**Published:** 2021-05-28

**Authors:** Thomas Gronwald, Sander Berk, Marco Altini, Laurent Mourot, Olaf Hoos, Bruce Rogers

**Affiliations:** ^1^Faculty of Health Sciences, Department of Performance, Neuroscience, Therapy and Health, MSH Medical School Hamburg, University of Applied Sciences and Medical University, Hamburg, Germany; ^2^Dutch Triathlon Federation, Arnhem, Netherlands; ^3^Faculty of Behavioral and Movement Sciences, Vrije Universiteit Amsterdam, Amsterdam, Netherlands; ^4^EA3920 Prognostic Factors and Regulatory Factors of Cardiac and Vascular Pathologies, Exercise Performance Health Innovation (EPHI) Platform, University of Bourgogne Franche-Comté, Besançon, France; ^5^Division for Physical Education, National Research Tomsk Polytechnic University, Tomsk, Russia; ^6^Center for Sports and Physical Education, Julius-Maximilians-University of Wuerzburg, Wuerzburg, Germany; ^7^College of Medicine, University of Central Florida, Orlando, FL, United States

**Keywords:** HRV, autonomic nervous system, endurance exercise, endurance training, polarized training

## Abstract

A non-linear heart rate variability (HRV) index based on fractal correlation properties called alpha1 of Detrended Fluctuation Analysis (DFA-alpha1), has been shown to change with endurance exercise intensity. Its unique advantage is that it provides information about current absolute exercise intensity without prior lactate or gas exchange testing. Therefore, real-time assessment of this metric during field conditions using a wearable monitoring device could directly provide a valuable exercise intensity distribution without prior laboratory testing for different applied field settings in endurance sports. Until of late no mobile based product could display DFA-alpha1 in real-time using off the shelf consumer products. Recently an app designed for iOS and Android devices, HRV Logger, was updated to assess DFA-alpha1 in real-time. This brief research report illustrates the potential merits of real-time monitoring of this metric for the purposes of aerobic threshold (AT) estimation and exercise intensity demarcation between low (zone 1) and moderate (zone 2) in a former Olympic triathlete. In a single-case feasibility study, three practically relevant scenarios were successfully evaluated in cycling, (1) estimation of a HRV threshold (HRVT) as an adequate proxy for AT using Kubios HRV software via a typical cycling stage test, (2) estimation of the HRVT during real-time monitoring using a cycling 6 min stage test, (3) a simulated 1 h training ride with enforcement of low intensity boundaries and real-time HRVT confirmation. This single-case field evaluation illustrates the potential of an easy-to-use and low cost real-time estimation of the aerobic threshold and exercise intensity distribution using fractal correlation properties of HRV. Furthermore, this approach may enhance the translation of science into endurance sports practice for future real-world settings.

## Introduction

Endurance exercise and training incorporates a wide range of intensity and duration, from several hours at low intensity to brief high intensity intervals lasting several seconds. These efforts are typically separated into three intensity zones delineated by certain physiologic thresholds. The first zone boundary (zone 1–2) is generally defined by the first lactate (LT1) or ventilatory threshold (VT1) as an aerobic threshold (AT; Meyer et al., 2005; Beneke et al., 2011; Hofmann and Tschakert, 2017; Bourgois et al., 2019). The second zone transition (zone 2–3) is felt to be related to concepts such as the second lactate threshold (LT2), maximum lactate steady state (MLSS), second ventilatory threshold (VT2) or respiratory compensation point (RCP) as an anaerobic threshold (Bourgois et al., 2019). Accurate and easy determinations of these thresholds would therefore be essential to establish and compare the various training intensity distribution strategies for fitness optimization and performance enhancement (Seiler and Kjerland, 2006; Treff et al., 2019).

Studies have attempted to show both the advantages and adverse consequences of time spent training at the various zone ratios. As an example of showing the importance of high volume and low intensity training, the volume of “easy runs” done by competitive long-distance runners was related to future performance more than the amount of high intensity training (Casado et al., 2019; Emig and Peltonen, 2020). In addition, of the various models that have been employed, such as polarized, threshold or pyramidal training intensity distribution, all have a common attribute, namely a large volume spent below the AT (Seiler and Kjerland, 2006; Esteve-Lanao et al., 2007; Stöggl and Sperlich, 2015, 2019; Bourgois et al., 2019). Although incremental exercise tests with measurement of lactate concentration and/or gas exchange are commonly done to define thresholds, the different approaches do not lead to the same results (Chicharro et al., 1997; Pallarés et al., 2016; Jamnick et al., 2020). In addition, commonly used metrics for VT1 (i.e., heart rate, HR) could be not accurate enough, e.g., in case of HR drift with dehydration for example. Failure to have precise definition of these boundaries could lead to undesirable training loads and unintentional distribution within the different intensity zones. The physical sequelae of even minimally exceeding a low intensity target may include delayed cardiac parasympathetic recovery (Seiler et al., 2007; Stanley et al., 2013), glycogen depletion (Beneke et al., 2011), gastrointestinal barrier disruption (van Wijck et al., 2012), along with more overall muscular and central fatigue (Noakes et al., 2005; Venhorst et al., 2018). All of this could ultimately lead to non-functional overreaching (Bourdon et al., 2017).

Given the complexities in easily defining markers for zone 1–2 transition, the question arises whether there are alternatives that could be derived via low cost, non-invasive, and commonly available wearable devices. Therefore, various indexes of heart rate variability (HRV; providing HR time series by RR-intervals) resulting from time- and frequency-domain analysis have been studied during dynamic exercise and have been shown to alter as work rates increase, with the greatest change occurring during lower intensities (Tulppo et al., 1996; Sandercock and Brodie, 2006; Karapetian et al., 2008; Michael et al., 2017). However, in response to the concern over loss of dynamic range past moderate exercise intensity (Persson and Wagner, 1996; Tulppo et al., 2005), non-linear methods of HRV analysis possess distinct advantages in providing new insights for training intensity distribution from a holistic autonomic nervous system perspective referred to as “physiological self-regulation” during endurance-typed exercise (Gronwald et al., 2020).

Recently, a study by Rogers et al. (2021a) showed good agreement between the exercise intensity reached at VT1 and a particular numeric value of a non-linear HRV index based on fractal correlation properties called alpha1 of Detrended Fluctuation Analysis (DFA-alpha1; Gronwald and Hoos, 2020). If we assume this relationship holds across a wide range of demographic groups, how can we make use of this property in day-to-day training and possibly support the translation of science into real-world sports practice (Fullagar et al., 2019; Coutts, 2020)? According to current data, as exercise intensity rises, DFA-alpha1 declines from values of well above 1.0 (fractal and well “correlated” patterns) during light exercise, passing 0.75 during the transition from low to moderate exercise intensities around AT with further dropping to below 0.5 (“uncorrelated,” random patterns) well beyond the AT (Gronwald et al., 2020). As the rate of change per work rate elevation seems highest near the AT and coincides with a DFA-alpha1 of 0.75 that delineates a trade-off between fractal correlated properties and uncorrelated randomness, this dimensionless index of overall physiological demands (Gronwald et al., 2019) bears the potential to provide real-world exercise intensity distribution without the need of an a priori normalization procedure using gas exchange data or blood lactate concentration. In other words, a DFA-alpha1 value below 0.75 during exercise would correspond to an intensity above the AT without prior knowledge of power, relative heart rate, blood lactate values, and ratings of perceived exertion (Jamnick et al., 2020), providing an opportunity to monitor real-time exercise intensity distribution for real-world endurance exercise and training settings.

Although HRV software packages like Kubios HRV (Tarvainen et al., 2014) can provide information of time spent below or above the AT by analyzing the recorded beat-to-beat patterns in RR-intervals from commonly worn HR monitor chest belts, it would do so in retrospect. In contrast, real-time monitoring of the DFA-alpha1 during an exercise session has several important benefits to an “after the fact” approach. These include:

- Information about global physiological demand and current absolute exercise intensity without prior formal lactate or gas exchange testing (e.g., exercise above or below the AT).
- Immediate estimation of HR/power output at AT using either an exercise stage test or constant power intervals done by cycling or running.
- Avoidance of post session data offload, processing, and interpretation using software packages dedicated to HRV analyses.

Recently, an app designed for iOS and Android devices, HRV Logger was updated to monitor real-time DFA-alpha1 every 2 min on screen while recording from commonly used HR monitors. Therefore, this single-case feasibility study aims to illustrate the potential merits of real-time assessment of this metric for the purposes of AT estimation as well as exercise intensity distribution and stimulate the translation of science into endurance sports practice for future real-world settings. It was hypothesized that: (1) a conventional retrospective analysis of an incremental cycling stage test performed by a former Olympic triathlete using Kubios HRV software via the method of Rogers et al. (2021a) yields a proxy of AT; (2) a cycling session using a series of constant power intervals represents a technique for real-world estimation of the AT based on DFA-alpha1 (HRVT) using the app, HRV Logger; (3) an AT confirmation and polarized training monitoring is possible in a typical 1 h cycling training session also using the app.

## Methods

### Participant

The participant was a former Olympic male triathlete (age 41, height 181 cm, weight 71 kg) who was in good health with no previous medical issues. Average training volume was 8 h per week (over the last year) consisting of a mixture of both low, moderate, and high intensity triathlon related training. As a reference, LT1 power was measured at ~220 W with HR at ~148 bpm based on recent incremental cycling stage lactate testing (start: 100 W, increment: 30 W, stage duration: 4 min). There were no regular medications, no tobacco, alcohol, caffeine or recreational drug use. The participant is an embedded sports scientist for elite performance athletes in triathlon to bridge the gap between sports science and coaching practice. The participant was informed about the case study procedures and objectives and provided written informed consent according to the ethical guidelines in accordance to the institutional review board and the guidelines of the Helsinki World Medical Association Declaration.

### Exercise Protocols

All three exercise protocols were performed with an interval of 1 week. Ambient temperature, meal timing, and cycling cadence were similar across all test conditions.

a) Classic stage test

An incremental cycling stage test (start: 80 W, increment: 20 W, stage duration: 4 min) until voluntary exhaustion was performed with a Cyclus2 ergometer (RBM elektronik-automation GmbH, Leipzig, Germany). Machine calibration was done in accordance with manufacturer recommendations.

b) 6 min stage test

An incremental cycling stage test (start: 160 W, increment: 30 W, stage duration: 6 min) was performed on the same Cyclus2 ergometer as the conventional stage test, but was modified to be applicable for road field tests by manually controlling power. Since the HRV Logger app displays a DFA-alpha1 value every 2 min, it was decided that constant power intervals start and end at increments of 2 min to optimally view data during elapsed interval time. In this example, after a 20 min warm up the participant started the first stage of the test at exactly 20 min with the 160 W stage (at a perceived “easy” level). At that point, a series of 6 min constant power intervals at 190, 220, and 250 W were performed with the test terminating at a submaximal intensity level. Since the first 2 min segment of the interval is not at a metabolic steady state, the first 2 min elapsed value of each stage was discarded. For real-time estimation of HRVT, the values at 4 and 6 min should be appropriate (Rogers, 2020). This progression of cycling power was continued until DFA-alpha1 passed through the 0.7 to 0.8 range which would signify the HRVT related intensity (Gronwald et al., 2020; Rogers et al., 2021a). Since DFA-alpha1 drops quickly past the AT, an additional stage higher is recommended to confirm the previously measured HRVT.

c) 1 h training session

A 1 h simulated training ride was performed on the same Cyclus2 ergometer, also applicable for road field tests by manually controlling power. Initially, a 30 min free form warm up was done at an intensity well below the AT. Then a series of three 6 min intervals at a cycling power corresponding to AT −20 W, AT, AT +20 W were performed, each separated by 4 min of active recovery periods at a HR level of around 130 bpm.

### RR Measurements and Calculation of DFA-alpha1 Derived Threshold

**Figure 1:**
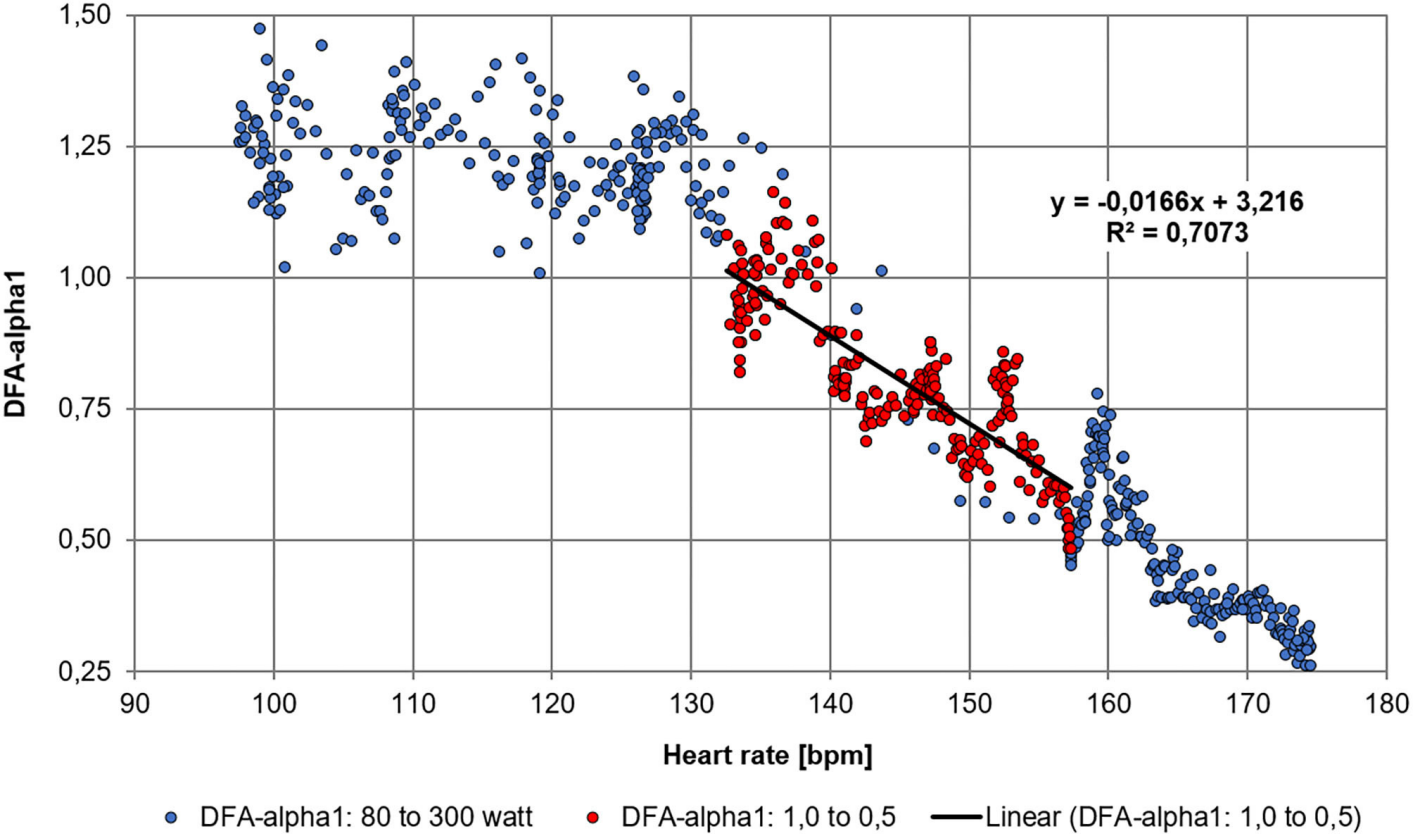
Time-varying results of DFA-alpha1 processed in Microsoft Excel with an equation for linear regression; DFA-alpha1 plotted against heart rate, the area of linear drop of DFA-alpha1 from 1.0 to 0.5 is used to determine the HR at HRVT, here at 148 bpm.

A Polar H10 (Polar Electro Oy, Kempele, Finland) HR monitoring device with a sample rate of 1000Hz was used to detect RR-intervals in all sessions. The RR-interval data was imported into Kubios HRV Software Version 3.4.3 (Biosignal Analysis and Medical Imaging Group, Department of Physics, University of Kuopio, Kuopio, Finland). Kubios preprocessing settings were at the default values including the RR detrending method which was kept at “Smoothn priors” (Lambda = 500; Tarvainen et al., 2014). DFA-alpha1 window width was set to 4 ≤ n ≤ 16 beats as in the original algorithm (Peng et al., 1995). Since HRV Logger app uses a correction technique similar to Kubios HRV medium threshold method, the RR-interval series was then corrected by the Kubios “medium threshold” method and relevant HRV parameters exported as text files for further analysis. Artifact levels measured by Kubios HRV were below 5%. DFA-alpha1 was calculated from the RR-intervals using 2-min time windows with repeat computation every 5 s throughout the test (time-varying method – window width = 2 min, grid interval = 5 s). Two-minute time windowing was chosen based on the calculations by Chen et al. (2002). For the detection of HRVT, a DFA-alpha1 value of 0.75 was chosen based on previous study in recreational athletes (Rogers et al., 2021a). This value is also the midpoint between a fractal, well-correlated behavior of the HR time series of 1.0 (seen with very light exercise intensity) and an uncorrelated value of 0.5 which represents white noise, random behavior (seen with high exercise intensity). Plotting of DFA-alpha1 vs. HR was then performed (see Figure 1), generally showing a sigmoidal decay curve with a stable area above 1.0 at low heart rates, a rapid, near linear drop reaching below 0.5 at higher heart rates and a probable flattening out close to maximum values. Plotting of DFA-alpha1 vs. HR over the span of DFA-alpha1 values between 1.0 and 0.5 should produce a relatively straight section. An equation for linear regression is obtained thereby allowing calculation of HR when DFA-alpha1 equals 0.75. The resulting HR is the HRVT as heart rate. For the calculation of cycling power at HRVT, an average of the 60 s power centered at the time DFA-alpha1 reaching 0.75 was chosen.

During both real-time assessment sessions, the recording of RR-intervals and display of DFA-alpha1 was done with an iPhone (via Bluetooth connection) using the HRV Logger app for iOS. The app provides a native implementation of DFA-alpha1 in Objective-C code. The implementation is also available as open-source code in python on GitHub (see Supplementary Material). App preferences were: “2 min measurement windows” and artifact correction was set to “workout mode” (similar to Kubios HRV “threshold” method). In addition, HR time series data was recorded simultaneously by a Garmin cycling computer (Edge 530, Garmin Ltd., Schaffhausen, Switzerland) with later Kubios HRV software processing for the purpose of a direct comparison to HRV Logger output.

## Results

a) Classic stage test: Baseline retrospective assessment of DFA-alpha1 derived threshold HRVT

Retrospective calculation of HRVT from the conventional stage test is presented in Figure 1 with HR yielding a value of 148 bpm. In addition, computation of HRVT as power was 214 W. The stage test was terminated at a power of 300 W.

b) 6 min stage test: Real-time assessment of DFA-alpha1 derived threshold HRVT

**Figure 2:**
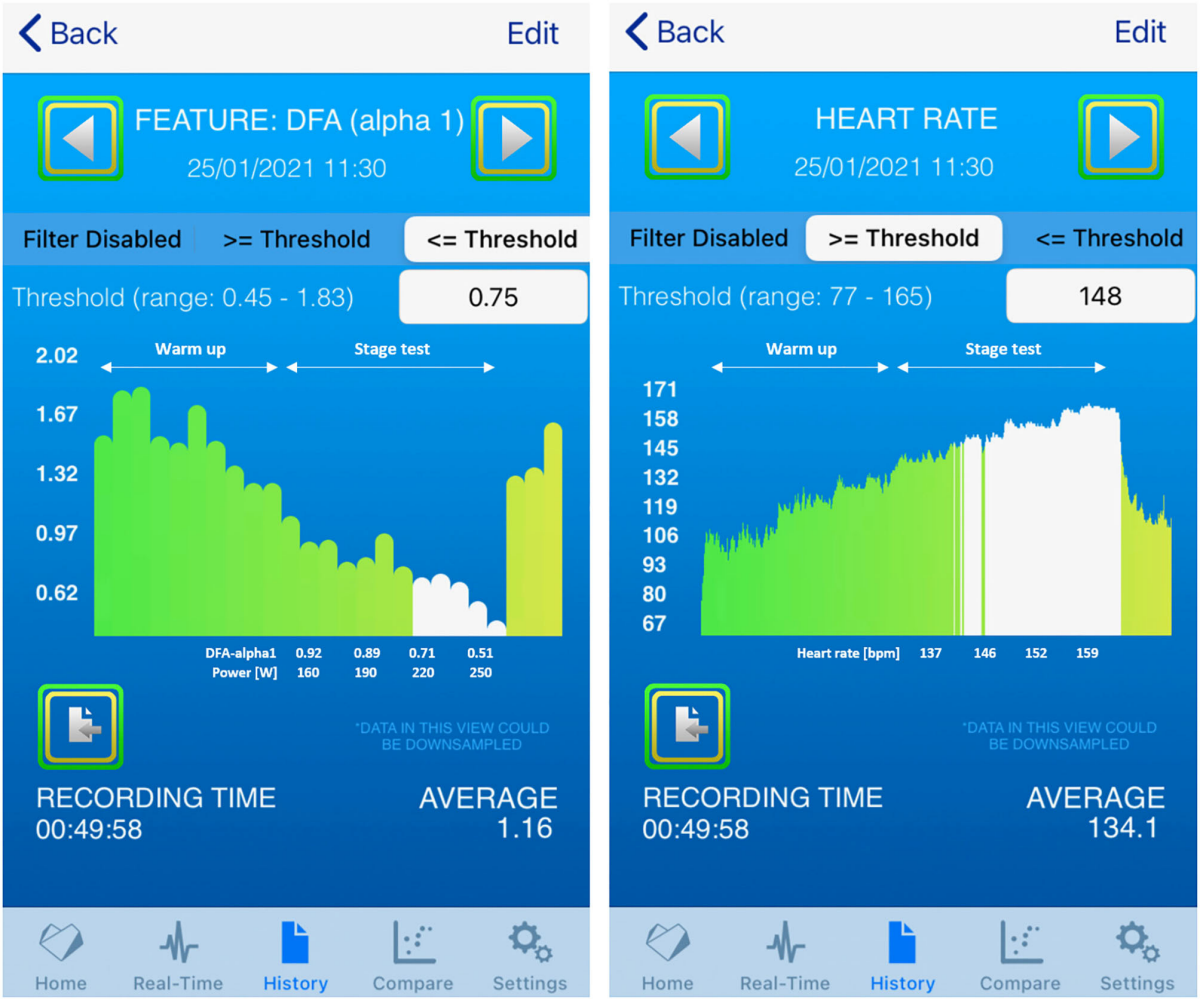
History view of the incremental 6 min stage test from 160 to 250 W and included values (left: DFA-alpha1 with power values, right: HR); DFA-alpha1 values displayed in white are the average of the two data points taken at 4 and 6 min.

**Figure 3:**
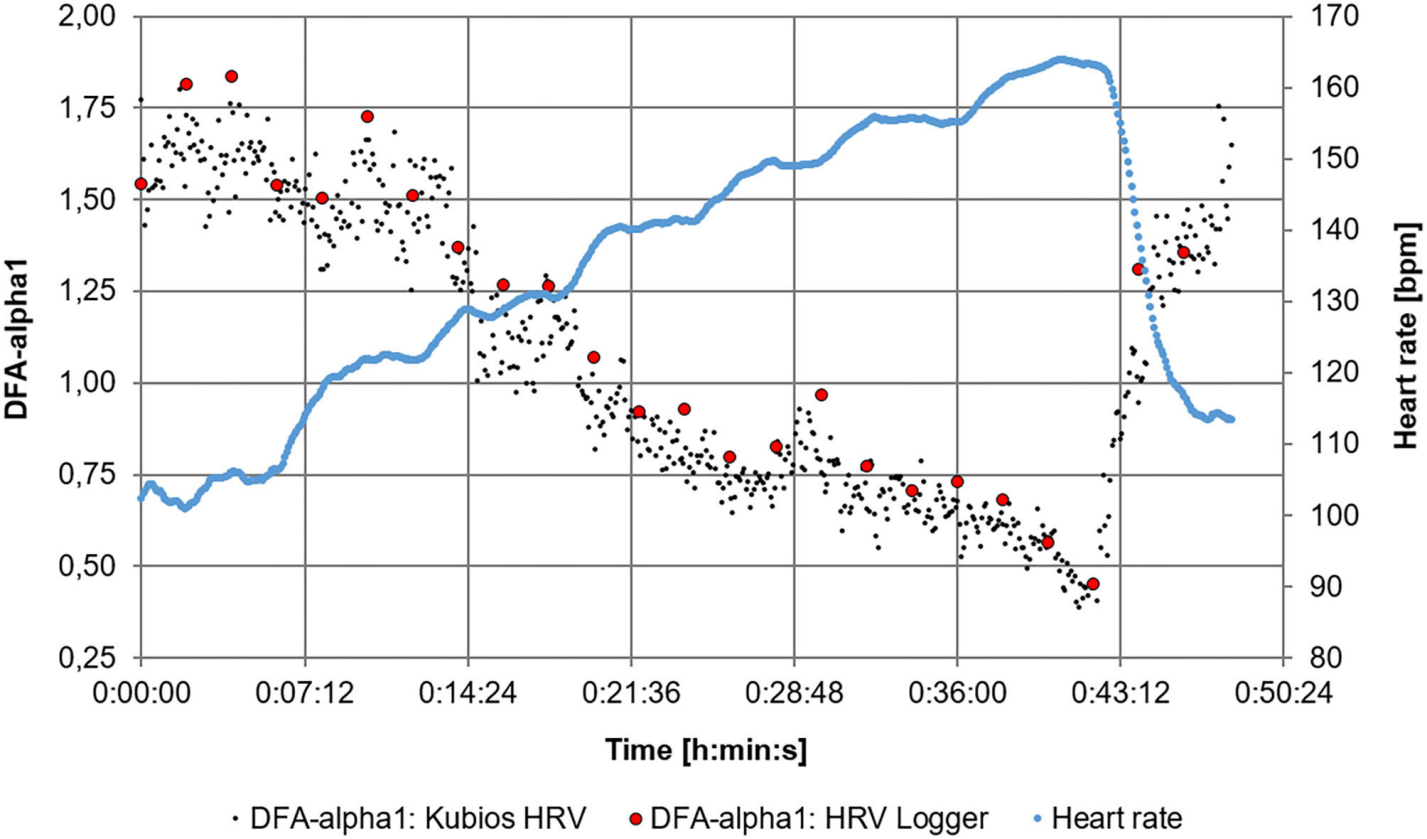
Comparison of DFA-alpha1 of the HRV Logger data export (2 min segments non-overlapped) and the raw data evaluation of the Garmin fit. file via Kubios HRV premium software (time-varying method, window width = 2 min, grid interval = 5 s) throughout the 6 min stage test.

Progressive decline in DFA-alpha1 occurred with increasing cycling power. DFA-alpha1 at 190 W was just below, 220 W close to, and at 250 W clearly above the HRVT as defined by a value of 0.75 (see Figure 2). To verify the validity of the app implementation, Figure 3 shows the comparison of the HRV Logger data export and a posteriori raw data evaluation of the Garmin.fit file via Kubios HRV processed in MS Excel.

c) 1 h training session: Real-time assessment of exercise intensity distribution

**Figure 4:**
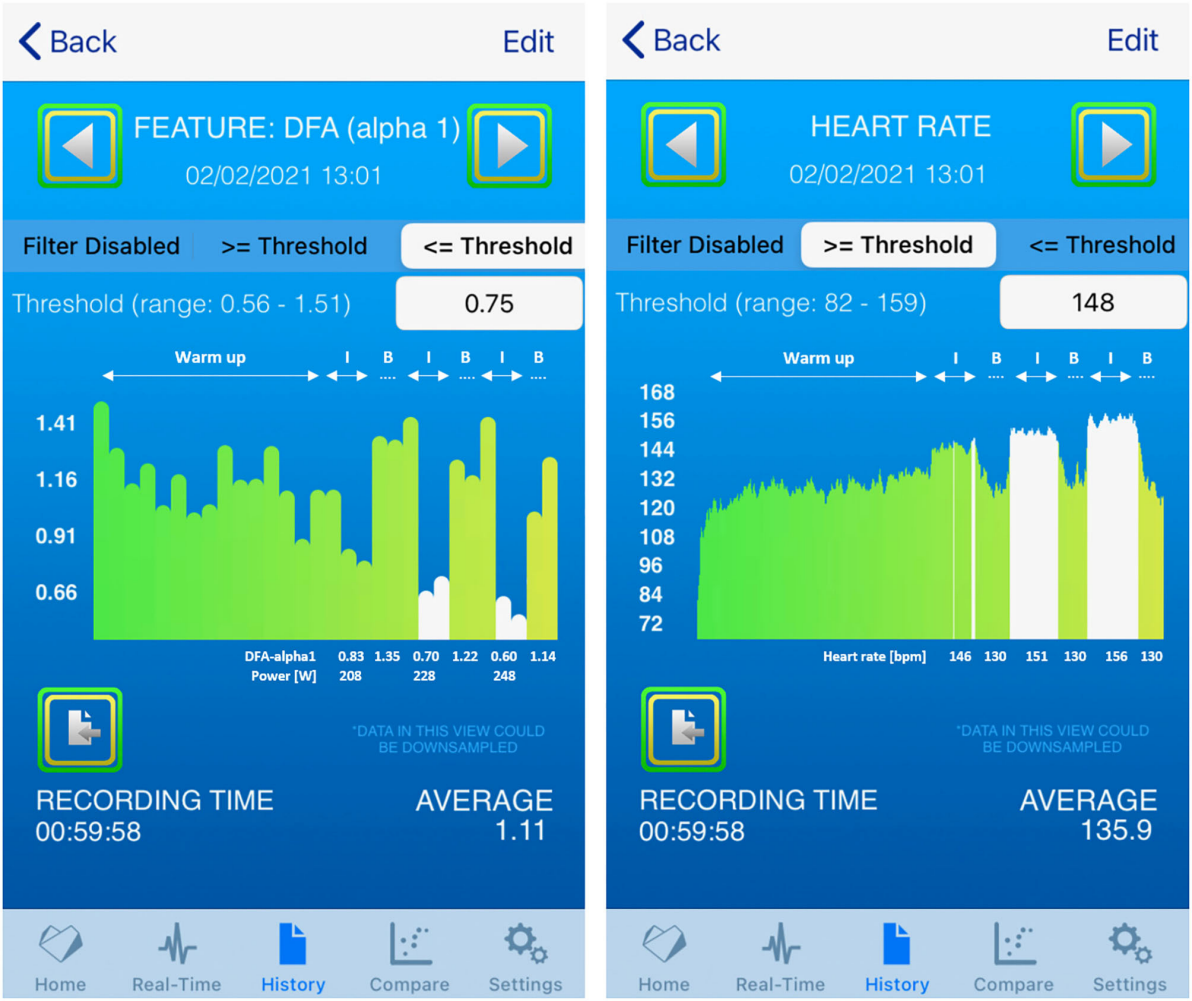
History view of the 1 h training session and included values (left: DFA-alpha1 with power values, right: HR); I: 6 min interval, B: HR based active recovery period; DFA-alpha1 values displayed in white are the average of the two data points taken at 4 and 6 min.

**Figure 5:**
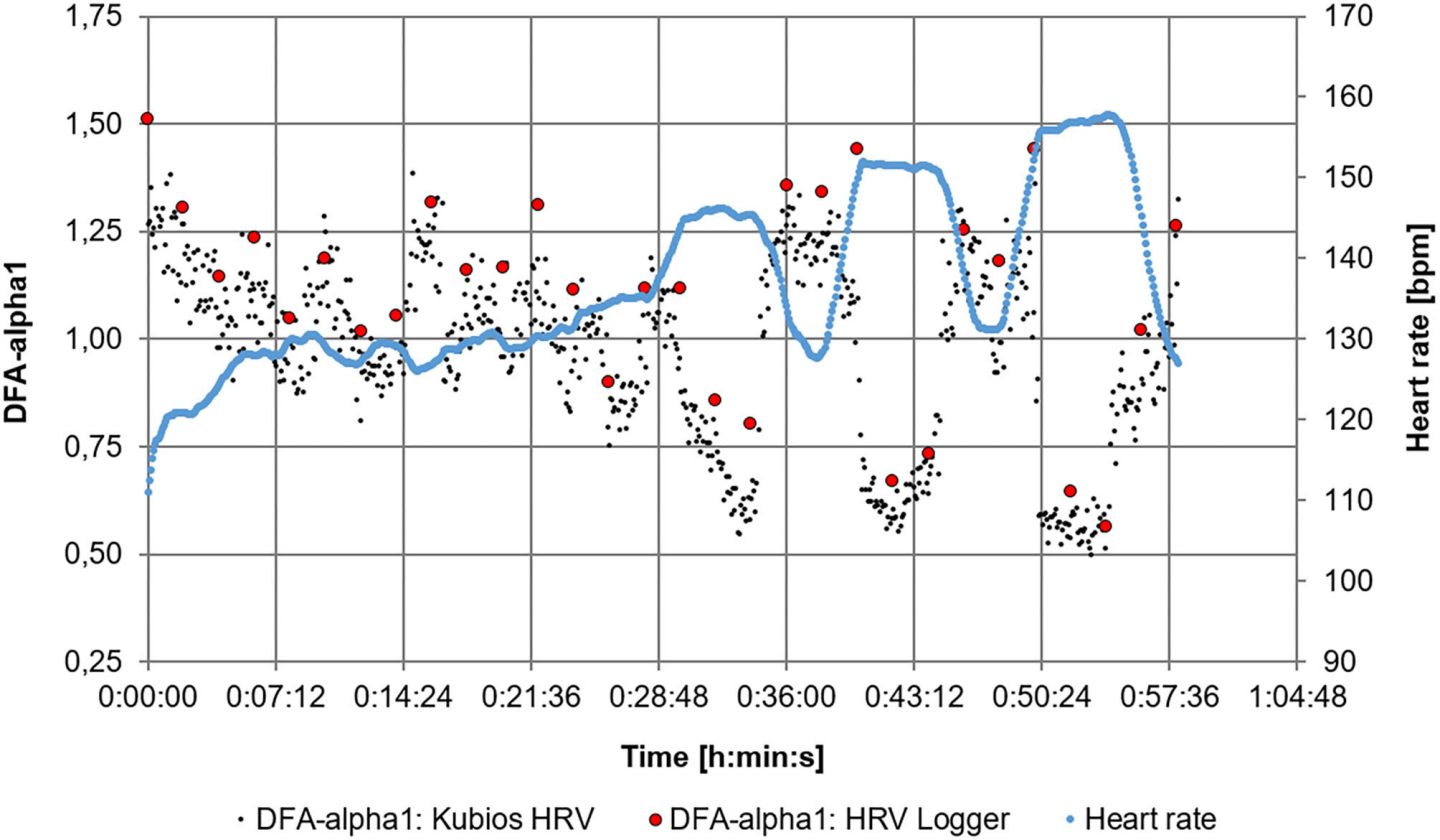
Comparison of DFA-alpha1 of the HRV Logger data export (2 min segments non-overlapped) and the raw data evaluation of the Garmin fit. file via Kubios HRV premium software (time-varying method, window width = 2 min, grid interval = 5 s) throughout the 1 h training session.

Average power for the 30 min warm up period of the 1 h training session was 143 W (65% of AT). The mean power for the three 6 min intervals were 208 W (mean HR: 146 bpm), 228 W (mean HR: 151 bpm), and 248 W (mean HR: 156 bpm). The first interval, done at below AT, displayed a DFA-alpha1 drop, but did not reach a value of 0.75 (0.83). The second interval, done at the AT associated power, displayed a DFA-alpha1 about 0.75 (0.70) and the final interval, done at above the AT related power, was well below 0.75 (0.60, see Figure 4). Figure 5 shows the comparison of the HRV Logger data export and a posteriori raw data evaluation of the Garmin.fit file via Kubios HRV processed in MS Excel.

## Discussion

The aim of this report was to demonstrate the potential advantages of real-time assessment of DFA-alpha1 by an inexpensive combination of readily available consumer grade products. Although not a formal validation study, the results presented here show reasonable clinical agreement between conventional retrospective calculation of the HRVT derived through Kubios HRV software to that of a real-time app, HRV logger in terms of power and heart rate. Results obtained by actively observing the DFA-alpha1 over sequential 6 min constant power intervals were easily understood by the participant and could be adapted to a purely field based test. Both power and HR at the intensity equaling DFA-alpha1 = 0.75 were similar in both the 6 min interval assessment as well as during the 1 h free form session showing the reliability of this metric. In addition, the recorded values fit well with the reference values of LT1 power at ~220 W with HR at ~148 bpm based on recent incremental cycling stage lactate testing. Since DFA-alpha1 is a dimensionless measure already normalized to an individual's internal load and low intensity boundary, the free form session itself could be used to estimate the AT as well as enforce an exercise intensity distribution plan.

Our intent was to outline a methodologic approach for future validation as well as illustrate the practical use of DFA-alpha1 to monitor internal exercise load in real-time athletic training settings providing translation of science into real-world practice (Fullagar et al., 2019; Coutts, 2020). In addition, since DFA-alpha1 is an index of physiological system demands it could provide further information as a global parameter for homeodynamic regulation and network physiology during endurance exercise (Balagué et al., 2020; Gronwald et al., 2020). Immediate feedback of current internal load before metabolic demand exceeds desired levels has obvious application to enforcement of the low intensity portion of a polarized or pyramidal training approach. In addition, zone boundaries may change due to seasonal factors (peaking and detraining), as well as current altitude level, temperature, humidity, and hydration status. As high volumes of low intensity training have been deemed critical for successful endurance performance, assessment of current physiological demands by DFA-alpha1 to assure compliance with the intended exercise zone 1 would be advantageous. Hence, DFA-alpha1 behavior during cycling activity in a former Olympic triathlete adds practical value to the previously validated concept of the HRVT in recreational runners (Rogers et al., 2021a). Hence, the provided approach can be considered as an original concept within the context of the theoretical systems dynamics framework in regard of different physiological regulation pattern (Gronwald et al., 2020), without the need for a priori normalization to gas exchange or blood lactate concentration (Jamnick et al., 2020). So even without prior knowledge of power, relative heart rate, blood lactate values, and ratings of perceived exertion, the real-time DFA-alpha1 approach provides an opportunity to monitor exercise intensity distribution in endurance-typed exercise and training. In addition, from the coaching perspective, the participant felt real-time assessment of DFA-alpha1 information could help in restricting training intensity to an intended zone 1 only session.

Another advantage of this approach is the avoidance of post training session data offload and processing. Adherence to a prescribed exercise intensity intervention would be made substantially easier without the time and effort needed to extract the relevant data through software such as Kubios HRV. Additionally, Kubios HRV requires a sizeable hardware footprint (4 GB of RAM, 3–5 GB of disk space, screen resolution of 1024 ×768 or higher, and the MATLAB Runtime installation) that may be an issue for many potential users. Although current smartwatch computational capability may not be sufficiently powerful enough for non-linear HRV processing, one could envision a custom smartphone app able to capture RR-intervals, appropriately process and present DFA-alpha1 on screen, as well as transmit the information to a smartwatch display.

## Limitations and Future Directions

Several issues could limit the application of this method to a broader population. The primary validation study for DFA-alpha1 as an AT surrogate was done in recreational male runners, making further investigation into other sports (e.g., cycling, XC ski, swimming, rowing) as well as with female participants necessary. Furthermore, DFA-alpha1 value derivation from the provided code has not been compared to that of Kubios software. Both preprocessing such as detrending method and artifact correction modalities are implemented slightly differently and could lead to potential bias. HRV missed beat artifact is common with moderate to high exercise intensity. Although we have shown that artifact rates below 5% when corrected by Kubios HRV should not have a significant effect on the HRVT (Rogers et al., 2021b), the HRV Logger app has not had similar validation but provides information about the current artifact rate in the latest version. Rogers et al. (2021b) has shown that uncorrelated DFA-alpha1 (0.5) is falsely elevated by artifact levels at 6%, potentially leading to under estimating intensity information. As opposed to a desktop PC implementation where a “grid interval” of 5 s is possible, the app is only able to provide a DFA-alpha1 value every 2 min, limiting a more granular estimation of DFA-alpha1 over time.

## Summary of Practical Recommendations for Endurance Exercise and Training

Based on the first validation study (Rogers et al., 2021a) of HRVT as well as one of artifact (correction) and device bias on DFA-alpha1 and HRVT (Rogers et al., 2021b) it is recommended to use a chest belt HR monitoring device such as a Polar H series with a sample rate of 1000 Hz. Retrospective approaches to determining the HRVT rely upon recording the RR-intervals with either a smartwatch, smartphone, or direct download from the HR monitor. For the retrospective analysis the HR time series should then be imported into a HRV software package such as Kubios HRV (Tarvainen et al., 2014) with the above mentioned preprocessing settings and recording recommendations. In regards to artifact correction via Kubios HRV software, either medium threshold or automatic mode is recommended (which is only available in the premium version). Although only available in the Premium version of Kubios HRV, 2 min time windows with repeat computation every 5 s throughout the test (time-varying method – window width = 2 min, grid interval = 5 s) is helpful for DFA-alpha1 visualization over time. For those with the free, standard version, 2 min measurement windows are still used but can be overlapped every 30 s to provide a more granular output of values. Although the premium version allows a direct text file download of all time-varying outputs, hand copying of pertinent values will need to be done with the standard version. Regarding artifact occurrence, it is recommended to reject data containing more than 5% artifact based on the previous investigation of the effects of missed beat artifact on the HRVT (Rogers et al., 2021b). Despite the implementation of artifact correction, it may be helpful to visually inspect the entire test recording in Kubios HRV to determine sample quality, and arrhythmia. For example, atrial ectopy could lead to significant deviation of DFA-alpha1 values. Additionally, regarding DFA-alpha1 assessment via HRV Logger app, we recommend using 2 min measuring window length with the artifact correction method in “workout mode.”

## Conclusions

Real-time monitoring of DFA-alpha1, a non-linear HRV index based on fractal correlation properties shows great potential for field assessment of the aerobic threshold with commonly available consumer products using the app, HRV Logger. In addition, since no normalization to conventional lactate or gas exchange markers is required, DFA-alpha1 behavior during endurance exercise can be used as a tool to specify intensity distribution at the zone 1–2 transition in a 3 zone model for the purpose of different training paradigms (e.g., polarized training). Immediate exercise intensity assessment has the important advantage of constraining a low intensity training zone before limits are exceeded. In addition, both post session processing time and software logistics are eliminated. This case report will hopefully encourage manufacturers of athletic oriented smart devices to include this functionality in their upcoming products.

## Data Availability Statement

The raw data supporting the conclusions of this article will be made available by the authors, without undue reservation.

## Ethics Statement

Ethical review and approval was not required for the study on human participants in accordance with the local legislation and institutional requirements. The patients/participants provided their written informed consent to participate in this study.

## Author Contributions

TG, BR, and SB conceived the study. TG and BR performed the data analysis and wrote the first draft of the article. All authors revised it critically for important intellectual content, final approval of the version to be published, and accountability for all aspects of the work.

## Conflict of Interest

MA is the developer of the Heart Rate Variability Logger application for iOS and Android. The remaining authors declare that the research was conducted in the absence of any commercial or financial relationships that could be construed as a potential conflict of interest.
